# When Young Children Grieve: Perspectives from Day Care Staff on Supporting Parents and Children through Illness and Loss

**DOI:** 10.1177/00302228231166803

**Published:** 2023-04-05

**Authors:** Martin Lytje, Atle Dyregrov

**Affiliations:** 1 Department of Center for Crisis Psychology, 1658 University of Bergen, Bergen¸ Norway; 2 Danish Cancer Society, Copenhagen, Denmark

**Keywords:** Preschool bereavement, day care, parental support, parents, staff, illness

## Abstract

This study explores how Danish day care institutions provide support to bereaved families based on accounts from staff members. Through eight focus groups, 23 employees from 8 day care institutions were interviewed. Subsequently, using thematic analysis, five themes were generated. These were: (1) coping with critical illness at the institution, (2) supporting parents at the time of death, (3) how day care institutions structured their response to illness and bereavement, (4) the staff’s support needs and (5) advice for other staff and parents in a similar situation. The study finds that when a life-threatening illness and/or death enters the life of a child who attends day care, staff have a strong belief that their role is to support both child and parent(s). However, staff often perceive this as a difficult task and express the need for more guidance on how to provide support.

## Introduction

Statistics suggests that in Denmark, annually 11,000 children between the ages of 0–6 are affected by a parent’s critical illness, with around 250 children experiencing the loss of either their mother or father (Lytje, 2021). Globally, the precise numbers are less well established, but Perlman and colleagues (2010) estimate that approximately 4% of all children will endure parental bereavement prior to reaching the age of 18.

Experiencing critical illness and bereavement at an early age, is associated with a range of negative risks. This includes psychological reactions such as fear, guilt, anger, dysphoria, regression (Dowdney, 2000; Gray et al., 2011; Holland, 2008), insomnia, intrusive thoughts, apathy (Bylund-Grenklo et al., 2016; Zisook et al., 2014) and post-traumatic stress. In addition, long-term consequences include difficulties with social relationships, educational attainment, increased mortality, and health (Dyregrov et al., 2022; Høeg et al., 2018; 2019; Li et al., 2014; Nielsen et al., 2012). While the loss itself can directly impact the mental health of children, it can also lead to changes in the family’s socioeconomic and health circumstances, further exacerbating the negative effects on mental health.

Following the loss of a parent, the surviving parent must contend with new financial and practical realities in addition to managing their own grief. Beyond increased health and psychological risks, the bereaved parent may also struggle with practical tasks (Granot, 2005; Park et al., 2021) and finding an appropriate way to talk to their children about the loss (Bosticco & Thompson, 2005; Dion, 2003; Weber et al., 2021). According to Dion (2003), women might be at particular risk of financial challenges, as they often have a lower income than men, while belonging to a social group with more resources may counteract the detrimental impact that a crisis may have (Prix & Erola, 2017).

Researchers (Berg et al., 2016; Cerel et al., 2006; Park et al., 2021) have found that parents may also struggle to provide support to their children following the loss of a partner. According to Granot (2005), studies have indicated that insufficiencies in care may persist for years. She attributed these deficiencies to issues faced by the parent, such as anxiety, fear, and other unresolved matters regarding the loss. Some authors (Luecken et al., 2009; Worden, 1996) have found that parental care may be the most significant mediating factor in reducing negative physical and mental risk factors. Saldinger et al. (2004) noted that child-centred parenting was associated with fewer post-bereavement symptoms amongst children.

The new family situation may require increased support from day care and school staff, as they spend significant time with the bereaved child. Whilst some research (e.g., Holland, 2008; Lytje, 2017a) has been conducted on how school staff could support bereaved children, few studies suggest how day care staff could play a supportive role.

A report by the Danish Cancer Society (2020) examined institutions’ bereavement response through interviews with 603 day care managers and stated that structured bereavement response plans (b-plans) were available at 87% of institutions. These plans provide basic guidelines for staff who must contend with life-threatening illness or bereavement and are developed by individual institutions; thus, they vary in size and scope. However, they tend to focus on practical tasks (e.g., what must be done) rather than *how* these tasks should be executed (e.g., how to talk to parents or the child (Lytje et al., 2021)).

This study aims to present perspectives on how Danish day care institutions provide support to bereaved families based on accounts from staff members. However, before exploring this, the article will quickly present the Danish day care system and its support role.

## Danish Day Care

Danish day care institutions can be classified into three categories: nurseries for children aged 0–3, kindergartens for children aged 3–6, and integrated institutions that cater to all age groups (Daginstitutioner, 2020). Danish day care institutions are offered to families by local municipalities and provide day care for children aged 0–6. Whilst parents pay 25% of operational costs, the government subsidises the remaining 75% (Daginstitutioner, 2020).

Nearly 96% of Danish children receive day care services in their early years. Although day care is free only for families with low income, it is subsidized by the Danish welfare system for all families. In addition to support staff such as cooks and cleaning personnel, day care institutions also employ three types of pedagogical staff: assistants, who may have no formal education or a 2-year non-university assistant degree Pædagogisk assistent, 2020 social educators, who possess a 3.5-year university-level degree in social education and specialize in addressing the developmental, educational, and social needs of children Københavns Professionshøjskole, 2018 and managers, who typically hold a social education degree.

## Methods

The present qualitative study employed a pragmatic design framework and utilized focus group interviews (FGIs). FGIs were employed due to their potential to facilitate interviewees in developing their opinions and investigations collaboratively, which may be harder to achieve on an individual basis (Kitzinger, 1995). It is acknowledged that experts in the area recommend a minimum of four participants in each interview session (Carlsen & Glenton, 2011). However, in this study, the group sizes varied between two and five participants. Nonetheless, since the main objective of the study was to discuss events at the day care institution as a group, the term FGI was utilized in this report, regardless of the number of participants involved. While the individual interview traditionally is the recommended approach for sensitive research, several authors (e.g. Guest et al., 2017; Kitzinger et al., 2000) have challenged the assumption that FGIs are unsuitable for such research. For instance, Guest et al. (2017) proposed that sensitive and personal revelations were more likely to occur when participants engaged in focus groups. Furthermore, by allowing participants to choose when to talk and when to be quiet, FGIs provide them with control over how many details to share. FGIs were thus considered a powerful and appropriate tool for the current qualitative study. The interviews followed a semi-structured guide that the interviewers could stray from if needed.

## Participants

During eight focus group sessions a total of 23 employees from 8 day care institutions were interviewed. They included a mix of assistants, social educators and managers from all five regions of Denmark. For the purposes of this study, participants were recruited through two methods:

  1. Through contacts in Danish municipalities, which forwarded information about   the study directly to day care institutions in their area.
  2. Through a database of institutions generated during previous research (The Danish   Cancer Society, 2020).

To qualify for the study, participants had to have experienced the death of a parent at their day care institution. Moreover, the loss should have occurred at least 6 months prior to the study but no longer than 3 years ago. This ensured that participants had experience providing post-bereavement support and had sufficient recall of this experience. Finally, a third criterion was that the children had to have been between ages two and six when bereavement occurred. All participants who were initially recruited also chose to participate in the interviews.

Since the study’s main objective was to understand the support needs of bereaved parents with children in day care and the challenges which arose for staff that provided this support, it included both participants who had experienced expected deaths (illness) and unexpected deaths (suicide). Table 1 provides information about each focus group.

**Table 1.** Participant Details.

Institution	Staff Members Who Participated in the Focus Group	Experienced Loss	Child’s Age at Time of Parental Loss and Gender
Institution 1	Social educator (I), social educator (II)	Father died from illness	Age 3 (boy)
Institution 2	Manager, social educator, assistant	Mother died from illness	Age 2 (boy)
Institution 3	Manager, social educator, assistant	Father died from illness	Age 3 (boy), age 3 (girl)
Institution 4	Manager, social educator (I), social educator (II)	Father died from suicide	Age 5 (boy)
Institution 5	Social educator (I), social educator (II)	Father died from suicide	Age 4 (boy)
Institution 6	Manager, social educator (I), social educator (II), social educator, social educator (III), assistant	Father died from illness	Age 5 (girl)
Institution 7	Manager, social educator	Father died from illness	Age 3 (girl)
Institution 8	Social educator (I), social educator (II)	Father died from illness	Age 4 (boy)

## Ethics

The study followed the ethical guidelines presented in the framework for good practice in counselling and psychotherapy (Bond & Griffin, 2013). These guidelines describe how to ensure that participants receive the necessary information to provide or revoke their consent and outline how to conduct interviews in a way that minimises participants' discomfort. Furthermore, the study followed the requirements set by the European General Data Protection Regulation (General Data Protection Regulation (GDPR) – Official Legal Text (n. d.)*.*

To present the data, all participants were anonymised and given aliases. Since the study was based at an independent non-governmental institution, it was subject to an internal ethics review before it began. In Denmark projects do not require approval from an external ethics committee as long as no biological material is collected (National Scientific Committee, 2020). To further protect the participants, post-interview support from Danish Cancer Society psychologists was available if needed.

## Procedure

To streamline participation and provide the comfort of a familiar location, the interviews were conducted at the day care institutions where the participants worked. Two experienced interviewers were tasked with conducting all interviews. They brought cake to the meetings to create an informal atmosphere in which to present the goal of the session. Then, they provided information about the participants' ethical rights and presented an ethical form to sign by all interviewees. Although a range of research questions were prepared, the interviews took the form of lightly moderated open discussions to enable free-flowing conversations. Therefore, the sessions focused on the areas that participants found to be most important. They lasted between 60 and 90 minutes and were audio recorded and transcribed. The interviews concluded with a conversation about participants' experiences with the study and a check-in of their feelings about participating.

## Data Analysis

To ensure that the study centred staff members' stories and perspectives, thematic analysis was chosen. Thematic analysis is a flexible approach, and its main strength is the provision of detailed accounts of complex data (Nowell et al., 2017). In this study, the data was analysed in six steps: (1) familiarisation with the data, (2) initial coding, (3) identification of themes, (4) review of themes, (5) definition of themes and (6) report writing. To address the criticism that thematic analysis is not a transparent method, an adapted version of the framework proposed by Braun and Clarke (2006) was used. All FGIs were conducted and analysed in Danish before selected quotations were translated into English, with a focus on meaning rather than one-to-one translation.

During the initial data review, 13 themes were identified. Five themes were related to how day care staff support parents: (1) coping with critical illness at the institution, (2) supporting parents at the time of death, (3) how day care institutions structured their response to illness and bereavement, (4) the staff’s support needs and (5) advice for other staff and parents in a similar situation. The remaining eight themes cantered on the experience of providing support for the bereaved child. These themes have been reported in a separate article by Lytje and Dyregrov (2021). The five themes associated with parental support and staff challenges were selected for this article. To conclude, all themes and quotation selections were reviewed and approved by a secondary researcher.

## Quality and Rigour

In thematic analysis, rigour is closely associated with the ability to conduct systematic and transparent data analysis (Braun & Clarke, 2006; Terry et al., 2017). To ensure that individual analysis steps were documented, QSR International’s NVivo 12 software was used. The primary data analysis was conducted by the first author, whilst a second researcher subsequently reviewed the initial analytical steps and final themes. Finally, the data were presented to and discussed with national and internal collaboration partners.

## Results

This section presents the insights gained from interviews with 23 staff members from 8 day care institutions. Six institutions experienced parental loss caused by illness, whilst two contended with parental loss caused by suicide. In addition, several institutions shared experiences of earlier bereavements during the interviews. Table 1 provides an overview of participants and the primary types of loss at each institution.

### Coping with Critical Illness at the Institution

Whilst two participating institutions experienced a sudden parental bereavement, the remainder reported a period of illness preceding the loss. Staff were notified about the illness in various ways. For some participants, this process was chaotic and occurred near the time of death. For others, the information was provided in a more orderly and timely manner. A social educator from Institution six explained how they were notified of the illness:

The parents actually flagged me and Lisa [a colleague] down, just after we concluded a conversation with another family. They told us about it in a very simple, polite and orderly fashion. They didn’t really know what was going to happen with [the ill father]. And to be honest, everyone kind of believed that things were going to be okay.

The participants' responses to parental illness and the type of support that they provided were highly dependent on the family and the extent to which they had included their child in the proceedings. Interviewees reported that, whilst the parents generally did include their children, they seldom believed that children understood the severity of the situation:

Odin knew – he knew his mom was ill and that the illness was called cancer. But he did not know that this was something you could die from. Because you might be so lucky that she doesn’t, right? The parents were great and talked to him about everything that was going on. He also went to the hospital with them. (Social educator, Institution 2).

However, not all children were included. At Institution 8, a social educator reported a different experience:

They were counting on the dad to recover. But as more time passed, the more we witnessed the illness change him. We could see the way that things were going, but it took two years before he died. But you know, Ali, he was not told until 2 weeks before. He knew that his dad was ill but not that he was going to die.

The participants also noted that the ill parent’s behaviour sometimes changed and could result in new support needs. At Institution 6, a social educator explained that they had become aware that the parent needed additional support when he picked up his child:

It was important to Mikkel that all the staff knew about his illness. When you pick up at 4 o’clock, it’s not always the same staff that are there. So, it was important that everyone knew he was really ill. You could see that clearly: he had no strength to climb the stairs, and he struggled to lift his hand to summon the elevator. So, the important thing to us was that he was with Villa when he picked her up from day care, that he did not have to spend energy trying to get a hat that we might have forgotten somewhere. He would sometimes ask us to do such things and, of course, we did. But I believe that if you had not known how ill he was, you would have wondered why we allowed him to push us around so much.

Whilst some parents needed considerable support, especially as they neared death, it was imperative for others to maintain as much normalcy as possible. At Institution 3, a social educator explained:

Some mornings, she indicated that ‘I don’t want to cry’. ‘Just say good morning to me and leave me alone’. All of us staff wanted to be empathetic and hear how she was doing and so forth, but sometimes she could not have that. Not everyone who suffers from cancer wants to be asked how they are doing constantly, especially when the answer is ‘not so well'.

Although death was a topic that all institutions found difficult to discuss, some chose to directly address it. At Institution 2, the staff did this by inviting the ill parent to a meeting. The social educator explained:

I chose to bring in an external consultant because I needed someone who could pick me up afterwards, but also to ensure that we covered everything that we needed to and that things did not escalate into a ‘crying contest'. It didn’t. Of course, I got tears in my eyes when we were sitting there. I told them that this is difficult for me. Everyone sitting around that table. Then, I would say, ‘In order to do the best I can for your boy and to help support you, there are some questions I need to ask you'. Then, we discussed everything, including how she wanted the other parents to be informed about her death. I remember her telling me, ‘I’m no drama queen'. She wanted the message to be short and to the point. Then, when she died, I went home and wrote, ‘In accordance with the wishes of [parent’s names], I am writing with regret to inform you that [parent] has died from a long illness'. That’s what she wanted the message to say.

Generally, the institutions that took pre-emptive measures and held meetings with the family reported these to be difficult for both the parents and staff. However, no one regretted them, and participants noted that they helped to prepare everyone for the inevitable.

### Supporting Parents at the Time of Death

Not all institutions experienced loss following a period of illness. In two cases, they had to contend with sudden death due to suicide. These led to a more chaotic situation due to the nature of the loss and the institution’s lack of time to prepare for it. At Institution 5, a social educator recounted how she was notified about a suicide:

I didn’t understand a word she was saying. The mom was crying. I didn’t understand what was going on at all. I knew something was wrong. She first signalled that he was ill, and at first I thought that it had something to do with cancer. Because she cried so much, I said, ‘You know what, why don’t we go into the office'. Also, because I needed a few seconds to try to process what it was that she was trying to tell me because I was not fully sure. When we entered the office, she told me, ‘He's dead'. That he'd fallen over and was dead. And I can't quite remember it, but it was actually a while into the conversation before she told me that he had committed suicide… She did not mention that to begin with. I thought that he had had a heart attack, but it turned out that he had hung himself.

In another sudden death situation, a social educator from Institution one noted that, in addition to having to support a traumatised family, the institution also had to protect them from the curiosity of other parents. In a small community, this became especially difficult:

The loss was receiving a lot of attention on social media. But we said nothing. And we did not find it to be okay that they were gossiping about it. Because that family was grieving. So, we were very clear. Being a professional, I think, means being very clear about that and setting the stage by saying, ‘These people are grieving, so all of us have to show respect and understanding for what they are going through'.

For all types of loss, expected or otherwise, one of the first decisions that the institution had to make was their role at the funeral. Generally, the staff felt that it was important to be part of this ritual, both as a show of support and to share an important event with the child that they cared for. A social educator from Institution three explained:

I called her and asked about the funeral: how they wanted it and whether we were allowed to attend. Were other parents from their playgroup welcome? They were very open, and all of that was okay. So, she quickly called me back with the time and told me that everyone who wanted to come was warmly welcome. I relayed that message to the other parents.

The staff’s role in supporting the surviving parent did not end after the funeral. At Institution 1, a social educator discussed her role as follows:

It was a feeling that you go from being a social educator who takes care of their children to stepping into the core of the family when something so difficult, so delicate, is being shared. It makes it an intense experience. So, I gave [the mother] lots of hugs. I listened and I asked about her children. And then I asked about how she was doing because I knew that she was struggling. So, we tried to deal with everything.

At Institution 3, the manager noted that additional support was needed, especially when the staff saw that the surviving parent was struggling:

After the funeral, things got really difficult for her. They had a very unstable income; they were independent shop owners, did not earn much and had no insurance. They had been living in a mansion that she could no longer afford. So, all of that came on top of her grief. She had to deal with the fact that, on top of losing their dad, her children were also going to lose their home. That affected her severely.

Whilst institutions tried their best to offer additional support and advice to the surviving parent, some staff noted that not all parents were interested in receiving this support. At Institution 8, a social educator discussed the uncertainty of the situation:

We asked the mother how we could support her. She did not want our help, so it was difficult. We also struggled to know exactly what to offer her. I honestly believe that, at that time, she was just not receptive to our efforts.

### How Day Care Institutions Structured their Response to Illness and Bereavement

As can be seen from the first two themes, day care institutions adopted a strong supportive role during the period leading up to and following the parental loss. All participants said that the institutions had a role to play and a responsibility in such situations. The manager from Institution seven explained:

Whether we have a responsibility? I would say we do, since her daughter attends our institution. And she [the daughter] deserves the very best conditions. So, I believe we also need to offer the best possible support to both of them. I believe that to be our duty.

One of the first actions that the institutions took after they learned about a critical illness or loss was to inform the staff and hold a general meeting. A manager from Institution four discussed this:

It was important for all of us to get together and discuss what was happening. That Alex got the care he needed during this difficult time. That [the staff] got support for the approach that they chose to take from the entire staff group. So that no discussions would happen later about whether things were being done the right way. That we agreed on the approach, and that this was a burden that we shouldered together.

The next step in the support efforts was to determine how to approach the surviving parent and the type of support that they might need. At Institution 4, a social educator explained:

I found it difficult to know how to reach out to the parents and what you are actually supposed to do. You know, forcing the parents to tell us what words they are using at home, I find that difficult. To try and help them find the right words, so they don’t just tell their child that ‘he is a little ill'. But I knew we had to, and the conversation went well. It was a difficult task, but it went well.

Three of the institutions that were unsure about how to approach the situation sought external expertise and support from their local municipality. However, the experience with support was mixed. One participant was happy with the support that their institution had received, whilst two felt that it had been inadequate. A manager from Institution seven explained:

There was this grief consultant in our municipality. I did not feel that [the support] was very good. I talked to her over the phone, and then she sent us some notes that she had taken by hand. It wasn’t professional. It just wasn’t good enough. In such a situation, you need them to come to you and offer what they can.

Whilst all institutions had bereavement response plans available, participants disagreed about their helpfulness. Some found the plans to be very helpful, whilst others found them to be inadequate. At Institution 3, a manager discussed the benefits of their plan:

It works. It kind of is always at the back of our minds. It's not that we constantly look at it, but we use it to check that we are doing the things that we need to do. Also, all parents are informed about our support plan before they admit their child here. They know that, if they start here, this is what we do if something happens. That it is not something you can choose to opt out of.

At Institution 8, a social educator expressed how they found that their plan was outdated and lacked the content necessary to handle the family’s support needs:

I was thinking, ‘What are we going to do?' Well, we have this bereavement response plan. I go to the office and tell our manager, ‘I need the [b-plan]'. Then, I got a single piece of paper. I told her, ‘This is useless. I don’t need to know that I have to send flowers for the funeral. I need to know how to deal with the family and the child'.

Whilst the main issue with the above plan was its quality, there was a general consensus amongst participants that b-plans were insufficient on their own. At Institution 2, a social educator explained: “It’s not enough to have a plan. You also need to hold the necessary meetings and allocate extra resources. In our situation, we also needed extra personnel”.

### The Staff’s Support Needs

Supporting a family through loss was a difficult endeavour for many staff members, particularly those who never had encountered such a situation before. At Institution 2, a social educator explained how the loss affected her on a personal level:

It was difficult. I cried a lot. But I managed to keep my professionalism. I also have a supportive husband at home. Often, I would sit with him and cry. However, I had the courage to be in that [supportive] role, even though it made me sad. Even now, as we are talking, I feel how I am still affected. But I am brave enough to cry, and these situations are so difficult to handle…

The extent to which staff felt prepared to cope with loss appeared to be highly influenced by whether they had previous experience with such situations. For those who did not, uncertainty about what to do and how to offer support was difficult. At Institution 8 a social educator explained:

We spent a lot of time searching for information on what to do. Because there was no one who told us how to deal with the situation. We lacked knowledge on how to support the mother. We needed to support the child, but we also needed to be there for the mother. But we simply lacked knowledge about how to support her and where else she might get help.

Regarding difficult conversations and considerations, participants generally noted the importance of having supportive colleagues. At Institution 2, an assistant expressed the importance of receiving support from colleagues:

We were good at talking things through. We also discussed everything at a staff meeting. Here, we dealt with all our worries: ‘I find this to be difficult', ‘what if. . . ', ‘I don’t know how to . . . '. So, all of the uncertainty, we talked it through. It was very helpful.

Whilst most staff coped well with the difficult experience with support from their manager and co-workers, others admitted to experiencing significant struggle. At Institution 1, a social educator discussed one situation that negatively affected her on a personal level. In this case, a child only had one parent who was critically ill:

I became very sad. And I took those feelings home with me. I did. I wasn't that professional back then. I ended up needing to see a psychologist because I struggled to define my role. I got wrapped up in the idea that, when the mom is dead, I have to take care of him. And then looking realistic at it. ‘No’, I'm not going to have to take care of him [The child] he has a family. My job is to take care of Simon when he is here in our care, and I need to concentrate on that. That gave me peace; I was his social educator, and that was the role I had.

This quotation exemplifies how difficult it could be for staff members to make a clear distinction between the professional and personal spheres in their support efforts.

### Advice for Other Staff and Parents in a Similar Situation

At the end of the FGI sessions, participants were asked about lessons learned from their experiences of loss at the workplace that they would like to share with other institutions. At Institution 5, a social educator highlighted the importance of being prepared for such situations before they happen:

You have to be on top of it. Have the courage to go through those considerations that you don’t want to. That someone might die or go through a very difficult time or that the mom might struggle to provide support. Whether to attend the funeral or not. You have to be willing to consider such things and say them aloud.

Similarly, a social educator from institution eight noted their struggle to determine what type of support to provide to the bereaved parent:

I wish that we had been better able to support the mom and tell her what we could actually offer by way of support. Then, she might not have turned us down. So, such things need to be written down. What we can offer and what we are allowed to offer.

A manager from institution seven proposed that, whilst the questions that followed illness and bereavement were difficult, they still needed to be asked:

It is important to be open, no matter how difficult the conversation is. You know, having to call a mother the day after her husband died, that is not fun. But it is a necessity. You have to – at least, that is what I believe. Because things can’t just be like they were before. Things have changed. She lost her husband, someone she loves, and the least you can do is ask her, ‘How are things, what do you need?'

A social educator at Institution two agreed with the importance of being open but also acknowledged that much of the grief work must be performed within the family at home:

We can only offer our support and advice. We cannot decide whether the family want to accept our support. However, we encourage them to be open about what has occurred and closely collaborate with us. We are the ones who need to reach out and say, ‘Let's talk about it'. We are the ones who have to encourage those difficult conversations.

All the participants felt that day care institutions had an important role to play in supporting both the bereaved child and the surviving parent.

## Discussion

Despite the availability of prior research exploring bereavement responses in schools (e.g. Holland, 2016; Lytje, 2018), our study represents a pioneering effort in examining bereavement responses in day care institutions. This study was able to leverage the insights garnered from previous studies conducted in educational settings, while acknowledging the disparities in the age and comprehension of the day care children and the varying levels of availability and training of staff. Nonetheless, the examination of bereavement responses in day care institutions has been made possible by drawing inspiration from prior research in related fields.

With respect to the central conclusions of this study, it is a noteworthy find that the Danish day care staff assumed the responsibility of providing support to families in crisis. They saw this as part of their role and were keen to be helpful and support both the child and the surviving parent. In fact, they went so far as to advocate for their presence at the funeral, with the intention of comprehending the child and the family’s cultural context.

Whilst some participating institutions struggled to know what to do or convince parents to accept their support, all of them stated that they did their best to support the family. Often, this led to significant frustration when staff encountered families who did not want their help or felt that they lacked the necessary knowledge to provide quality support. Whilst participants generally stated that the bereaved child was their primary concern, they argued that they could not be helped without helping the parent, i.e., provide guidance on how to talk to children and protect from the curiosity of other parents.

The fact that day care staff feel that they have a role to play in supporting families through illness and bereavement is not a novel find. Both Scandinavian (e.g. Dyregrov et al., 2015; Lytje, 2013) and international (e.g. Costelloe et al., 2020; DeMuth et al., 2020) researchers have reported that school staff believe that they have a role to play in offering support in the time that follows a loss, although teachers can find this difficult (Dyregrov et al., 2013). This was confirmed by Holland (2001), who found that some British teachers struggled to know what to do following parental bereavement and sometimes took no action at all because they feared that they might cause pupils further pain.

Whilst Holland’s (2001) finds were related to students and not parents, the results of the current study indicate that childcare staff also find the provision of support to the family during and after parental illness and after death to be difficult. Indeed, this has also been noted by other authors (e.g. Galende, 2015; Herrero et al., 2020). However, in contrast to Holland’s (2001) findings, Danish day care staff seemed to acknowledge that it is sometimes necessary to push through their own discomfort to ensure that they provide the necessary support to children and their families.

Several factors may explain this engagement amongst day care staff. A likely explanation is that many staff members are professionally trained social educators. Although the duration of teachers' education is identical, social educators and teachers have different responsibilities. Whilst teachers aim to help children learn new skills, the main role of social educators is to ensure that children develop mentally, socially and physically Københavns Professionshøjskole, 2018a, 2018b. This appears to lead to a strong focus on assisting families through the grieving process and reintegrating bereaved children into day care in a positive manner. Thus, the presence of adequately educated childcare staff can safeguard children’s well-being.

Day care staff have a ‘physical’ relationship to the children that they care for; they often have them on their laps and individually interact with them during physical play, and the children are reliant on them for their daily needs. This creates a closer and more intimate relationship between the child and the day care staff and a closer psychological relationship than is typical between teachers and students. This may lead day care staff to feel more responsibility for individual children and make them more inclined to play a supportive role when they experience serious illness and loss. A natural part of this process is supporting parents in helping their child. However, day care staff may also experience considerable emotional pain when a child that they care for loses a loved one.

Another possible explanation for engagement amongst day care staff is the availability of structured b-plans at many institutions. A recent Danish Cancer Society report (2020) based on interviews with 603 Danish day care managers found that 87% of institutions had such a plan. Of these, 91% highlighted that knowing that b-plans were available gave the institution a sense of security, whilst 77% found that they improved the institution’s ability to respond to families in crisis. However, it must also be acknowledged that 40% of respondents felt that they needed additional training and tools to respond to bereavement (ibid). Nonetheless, even if some b-plans may be inadequate, they still serve as a ‘written contract’ that highlight bereavement as something the institution chose to address. Having plans meant that they could take pre-emptive measures and mount a structured response. Having previous experience meant less uncertainty about what to do. Uncertainty was also higher following sudden losses where external help was more sought out. In regard to the literature, while no articles exist which offers guidelines on how to support day care children, such articles do exist in relation to schools (e.g., Dyregrov et al., 2020; Holland, 2008).

### Conceptualisation of Grief Work

This study provides perspectives on how day care staff perceive ‘good’ bereavement support and grief work. It is notable that participants highlighted their institutions' efforts to help the child and their family process their grief. They recommended forms of support that provided opportunities for the child to express their feelings about the loss and share their grief. Whilst many participants reported that their institutions focused on ensuring that the family could experience a ‘normal’ day, none believed that it was best to not discuss or hide the difficult experience. Instead, they usually ensured that the day care community addressed the loss together. This was reflected in the messages that institutions sent to all parents and their help with coordinating participation at the funeral.

### Consequences for Supporting Staff

Based on the accounts provided in this study, it is clear that whilst all staff acted, some were more active than others. Several participants reported feeling that the role of supporter was stressful and entailed personal costs. In fact, a few were so severely affected that they needed external support in the form of either an expert on grief or a psychologist to help them face their own fears and feelings of inadequacy.

In a small-scale study, Lane et al. (2014) uncovered similar issues amongst British teachers. The interviewees reported feeling that they needed to stay strong to provide support to bereaved students, even at a personal cost. A more recent study by Costelloe et al. (2020), who interviewed 16 British teachers, also found that participants had a sense of duty to provide support. The authors noted that the provision of this support had a negative impact on the emotional well-being of teachers, with the most frequent feelings related to stress, worry, guilt and sadness (Costelloe et al., 2020). This issue was so widespread that the authors emphasised that educational psychologists embedded in schools must offer support not only to the bereaved child but also to supporting teachers. In the current study, participants expressed similar feelings. It was already noted that the relationship between children and day care staff is emotionally and physically closer; thus, supporting staff may require greater attention. Good support may both help the staff in interactions with children and parents and prevent burnout (Ishibashi et al., 2022).

## Limitations

Since this study is both qualitative and exploratory in nature, the findings must be treated with caution. In most of the interviews, day care managers participated on equal terms with other staff. This was logical, as they coordinated many parts of the institutions' bereavement response. However, a disadvantage is that other staff may have omitted views or stories that they did not want their managers to hear. The small groups involved may have increased a sense of vulnerability when sharing. Small groups may have made it easier for some, while creating more unease for others. Moreover, institutions that were proud of their support efforts may have been more likely to participate in the study than institutions that were less proud of their response.

## Recommendations

This study has revealed that offering support to bereaved children and their families can be a complex and demanding task. Although having a pre-determined plan for providing such support may not always result in easier support efforts, issues encountered by staff often seemed to be due to shortcomings in the plan rather than the effectiveness of the planned approach. Moreover, the study highlights that active engagement of the family in support efforts is more beneficial than leaving them to their own devices.

Findings suggest that staff struggles are primarily encountered when problems arise that are not covered by the plan. Although no plan can account for every eventuality, the general issues experienced by staff indicate that day care institutions should focus on five key areas of preparation:

1. Family collaboration: Having established guidelines for contacting the family, arranging a meeting, and asking crucial questions can aid in structuring the support process.
2. Community involvement: Difficulties often arise when determining who to inform and how to engage with the community involved in the day care. Having prepared guidelines for these actions can mitigate such problems.
3. Staff support: Providing support to bereaved children and their families is a taxing task and can place a significant burden on primary care staff. Day care institutions should not only prepare for how to support the bereaved family but also ensure that primary care staff receive adequate guidance and support to cope with this task.
4. Facilitating difficult conversations: Addressing loss is difficult, but it is imperative for fostering a common understanding of the situation and for collaboration between the family and day care institution in supporting the grieving child.
5. Long-term perspective: It is essential to acknowledge that support should not be limited to the initial weeks after a loss and that day care institutions must provide guidance and support as long as the child is in their care.

## Conclusion

In the event of a life-threatening illness or death affecting a child enrolled in day care, the staff members understand their role as providing support to both the child and their parent(s). They employ available b-plans and strive to create a supportive environment for the grieving child (Lytje et al., 2021). Despite recognizing the importance of their role, staff members often find this task taxing and express a need for more guidance on effective support provision.

Additional research to better understand the impact of parental illness and death on day care staff and their supportive role is welcome, as well as larger sample sizes to examine the complex relationship between the child, parent(s), and day care staff in the aftermath of loss. Given the potential difficulties faced by the remaining parent, it is crucial that day care staff possess the necessary skills, knowledge, and motivation to provide adequate support during this challenging time.
